# Severe Respiratory Disease Among Children With and Without Medical Complexity During the COVID-19 Pandemic

**DOI:** 10.1001/jamanetworkopen.2023.43318

**Published:** 2023-11-14

**Authors:** Christina Belza, Eleanor Pullenayegum, Katherine E. Nelson, Kazuyoshi Aoyama, Longdi Fu, Francine Buchanan, Sanober Diaz, Ori Goldberg, Astrid Guttmann, Charlotte Moore Hepburn, Sanjay Mahant, Rachel Martens, Apsara Nathwani, Natasha R. Saunders, Eyal Cohen

**Affiliations:** 1 The Hospital for Sick Children, Toronto, Ontario, Canada; 2 Edwin S.H. Leong Centre for Healthy Children, University of Toronto, Toronto, Ontario, Canada; 3 Child Health Evaluative Sciences, The Hospital for Sick Children, Toronto, Ontario, Canada; 4 Dalla Lana School of Public Health, The University of Toronto, Toronto, Ontario, Canada; 5 Department of Pediatrics, University of Toronto, Toronto, Ontario, Canada; 6 ICES, Toronto, Ontario, Canada; 7 Temerty Faculty of Medicine, University of Toronto, Toronto, Ontario, Canada; 8 Institute of Health, Policy, Management and Evaluation, The University of Toronto, Toronto, Ontario, Canada; 9 Department of Anesthesiology and Pain Medicine. The Hospital for Sick Children, Toronto, Ontario, Canada; 10 Institute of Medical Science, The University of Toronto, Toronto, Ontario, Canada; 11 Provincial Council for Maternal and Child Health; 12 Pulmonology Institute, Schneider Children’s Medical Center of Israel, Petach Tikva, Israel; 13 Faculty of Medicine, Tel-Aviv University, Tel-Aviv, Israel; 14 McMaster University, Canada

## Abstract

**Importance:**

Severe respiratory disease declined during the COVID-19 pandemic, partially due to decreased circulation of respiratory pathogens. However, the outcomes of children with higher risk have not been described using population-based data.

**Objective:**

To compare respiratory-related hospitalizations, intensive care unit (ICU) admissions, and mortality during the pandemic vs prepandemic, among children with medical complexity (CMC) and without medical complexity (non-CMC).

**Design, Setting, and Participants:**

This population-based repeated cross-sectional study used Canadian health administrative data of children aged younger than 18 years in community and pediatric hospitals during a pandemic period (April 1, 2020, to February 28, 2022) compared with a 3-year prepandemic period (April 1, 2017, to March 31, 2020). The pandemic period was analyzed separately for year 1 (April 1, 2020, to March 31, 2021) and year 2 (April 1, 2021, to February 28, 2022). Statistical analysis was performed from October 2022 to April 2023.

**Main Outcomes and Measures:**

Respiratory-related hospitalizations, ICU admissions, and mortality before and during the pandemic among CMC and non-CMC.

**Results:**

A total of 139 078 respiratory hospitalizations (29 461 respiratory hospitalizations for CMC and 109 617 for non-CMC) occurred during the study period. Among CMC, there were fewer respiratory hospitalizations in both 2020 (rate ratio [RR], 0.44 [95% CI, 0.42-0.46]) and 2021 (RR, 0.55 [95% CI, 0.51-0.62]) compared with the prepandemic period. Among non-CMC, there was an even larger relative reduction in respiratory hospitalizations in 2020 (RR, 0.18 [95% CI, 0.17-0.19]) and a similar reduction in 2021 (RR, 0.55 [95% CI, 0.54-0.56]), compared with the prepandemic period. Reductions in ICU admissions for respiratory illness followed a similar pattern for CMC (2020: RR, 0.56 [95% CI, 0.53-0.59]; 2021: RR, 0.66 [95% CI, 0.63-0.70]) and non-CMC (2020: RR, 0.22 [95% CI, 0.20-0.24]; RR, 0.65 [95% CI, 0.61-0.69]). In-hospital mortality for these conditions decreased among CMC in both 2020 (RR, 0.63 [95% CI, 0.51-0.77]) and 2021 (RR, 0.72 [95% CI, 0.59-0.87]).

**Conclusions and Relevance:**

This cross-sectional study found a substantial decrease in severe respiratory disease resulting in hospitalizations, ICU admissions, and mortality during the first 2 years of the pandemic compared with the 3 prepandemic years. These findings suggest that future evaluations of the effect of public health interventions aimed at reducing circulating respiratory pathogens during nonpandemic periods of increased respiratory illness may be warranted.

## Key Points

**Question:**

Did rates and outcomes of severe respiratory illness change during the first 2 years of the pandemic, compared with prepandemic, among children with medical complexity and those without medical complexity?

**Findings:**

In this repeated cross-sectional study of 139 078 respiratory hospitalizations in Canada, there were more than 45 000 fewer respiratory hospitalizations, more than 4200 fewer respiratory intensive care unit admissions and, among children with medical complexity, 119 fewer deaths during respiratory hospitalizations than expected in the first 2 years of the pandemic.

**Meaning:**

This study’s results suggest the need for evaluation of the effect of public health interventions in reducing circulating respiratory pathogens during nonpandemic periods.

## Teaser

This cross-sectional study compares respiratory-related hospitalizations, intensive care unit admissions, and mortality during the pandemic vs prepandemic among children with and without medical complexity.

## Introduction

The onset of the COVID-19 pandemic and the public health measures instituted to mitigate its spread were associated with a dramatic reduction in circulating respiratory viruses such as respiratory syncytial virus and influenza.^1,2,3^ Infections with respiratory viruses are common contributors to pediatric hospitalizations, either directly (eg, pneumonia) or indirectly (eg, asthma exacerbation).^4,5,6^ Dramatic reductions in pediatric health care use were noted during the pandemic, due at least in part to the decrease in respiratory viral infections.^7,8,9,10,11^ Children with medical complexity (CMC) are at risk of severe acute illness from respiratory infections (eg, children with cystic fibrosis,^12^ congenital heart disease,^13^ or sickle cell disease^14^). In a Canadian evaluation, children who were admitted with SARS-CoV-2 infections during the early pandemic period often had existing comorbidities including obesity and neurologic impairment.^15^ Children with neurologic impairment (NI), which account for 28% of all Canadian CMC,^16^ are at particularly high risk due to a number of factors, including impaired cough and airway clearance, respiratory muscle weakness, bronchial hyperactivity, sleep disordered breathing, and risk of aspiration from oral secretions.^17^ This may result in CMC having an outsized benefit from this general decrease in burden of circulating viruses.^18^ Reports from multiple countries have suggested decreased emergency department visits^7,8^ and admissions to hospital during the pandemic both for children with and without medical complexity,^9,10,11^ but these findings were limited to those reporting solely on children’s hospitals, evaluations during the early pandemic period, and were not denominated on a defined at-risk population. The effect of pandemic-era suppressed respiratory viral transmission on hospitalization, intensive care unit (ICU) admission, and mortality among CMC and children without medical complexity (non-CMC) is unknown.

Understanding the association of the pandemic with health care utilization related to respiratory illnesses among CMC and non-CMC in Canada, a country that instituted relatively stringent public health measures over the first 2 years of the pandemic,^19^ may inform our understanding of the potential benefits of nonpharmaceutical interventions (such as masking,^20^ reducing contacts,^20^ social distancing,^21^ and air filtration and purification^22^) aimed at protecting children at risk of respiratory hospitalizations during seasonal respiratory viral surges (eTable 1 in Supplement 1). Our objective was to evaluate changes in respiratory hospitalizations, ICU admission, and mortality among CMC and non-CMC during the pandemic compared with prepandemic. We hypothesized that there would be a larger decrease in severe respiratory hospitalization and ICU admissions among CMC compared with non-CMC, reflecting the use of nonpharmaceutical interventions mitigating illness transmission for those particularly at risk for infections.

## Methods

### Study Design and Population

This cross-sectional study used a repeated, population-based analysis and followed the Reporting of Studies Conducted Using Observational Routinely-Collected Data (RECORD) reporting guideline.^23^ Using data from the Canadian Institutes for Health Information Discharge Abstract Database (CIHI-DAD) between April 1, 2017, and February 28, 2022, we identified all non-newborn hospitalizations to every acute care hospital in Canada (excluding Québec, which accounts for 21% of Canada’s population) among children younger than 18 years of age (eTable 2 in Supplement 1). We included all respiratory hospitalizations using the Pediatric Clinical Classification System (PECCS), which categorizes common reasons for hospitalizations into clinically meaningful groupings^24,25,26^ (eTable 3 in Supplement 1). CMC were identified using the CIHI CMC methodology based on the Feudtner complex chronic condition (CCC) list,^14^ adapted for use in Canada^16,27^ using the *International Statistical Classification of Diseases and Related Health Problems, Tenth Revision, Canadian Edition (ICD-10-CA)* diagnostic codes and supplemented with high-intensity NI codes.^28^ A CMC hospitalization was defined as a child with any CCC or NI diagnosis code recorded in the 5 years before the index hospitalization.^16,27^ The population of children in Canada (excluding Québec) at the beginning of each year was obtained from Statistics Canada.^29^ Assuming temporal stability in the published proportion of Canadian children with CMC (948 per 100 000),^27^ we calculated CMC prevalence based on each year’s total pediatric population.

This study received ethics approval from the Hospital for Sick Children research ethics board. Waiver of consent was granted by the research ethics board due to the use of administrative data.

### Pre–COVID-19 and COVID-19 Periods

A prepandemic period (April 1, 2017, to March 1, 2020) was used to derive expected hospitalizations, accounting for time trends and seasonality. The pandemic period was divided into two 12-month periods corresponding with the public sector fiscal year (FY) in Canada (April 1 to March 31). We excluded a 1-month washout period (March 2020) at the start of the pandemic, defining the pandemic period as April 1, 2020, to February 28, 2022. Hospitalization data at CIHI is only captured at discharge, so we excluded the last month (March) of FY 2022 to minimize right-censoring.

### Variables

We identified all hospitalizations among CMC, which we further described based on 4 mutually exclusive diagnostic groups: NI and at least 1 CCC, multiple organ CCC (excluding NI), NI alone, and 1 non-NI CCC. We also identified hospitalizations for respiratory illnesses among children without medical complexity. For each hospitalization, we described hospitalization-level characteristics (length of stay in days, respiratory etiology subdivided into infectious vs noninfectious [eTable 4 in Supplement 1], ICU admission, province or territory of hospitalization) and child-level characteristics (sex, age category, mortality, diagnosis code for medical technology [eg, feeding tube^13^]). Race and ethnicity data were not available in the CIHI-DAD.

### Statistical Analysis

We evaluated changes in CMC and non-CMC respiratory hospitalizations, ICU admission, and in-hospital mortality, using a negative binomial regression model comparing prepandemic observed vs pandemic expected weekly event counts, and summarized as rate ratios (RR) by FY 2020 and 2021 offset by the total pediatric population each year. We conducted a sensitivity analysis limiting respiratory hospitalizations for CMC and non-CMC and mortality for CMC to those with PECCS codes corresponding to a clear infectious etiology (eg, bronchiolitis). As sex and age of the child can be associated with illness severity,^30,31^ we completed an additional analysis stratified on these variables. We assumed that nonoverlapping 95% CIs for group estimates indicated significant differences. All analyses were completed using SAS studio version 9.4 (SAS Institute) from October 2022 to April 2023.

## Results

There were 139 078 respiratory hospitalizations (29 461 for CMC and 109 617 for non-CMC) from March 1, 2017, to February 28, 2022 (Table 1). Children younger than 2 years of age were hospitalized most frequently, accounting for 10 271 (34.8%) of CMC and 56 652 (51.7%) of non-CMC hospitalizations. Male children accounted for the majority of respiratory hospitalizations for both CMC (16 291 [55.3%]) and non-CMC (63 659 [58.1%]). The length of stay among CMC was a median (IQR) of 5 (2-12) days for FY 2017 to FY 2019, 6 (2-18) days in FY 2020, and 5 (2-14) days in FY 2021. For non-CMC, the median (IQR) length of stay remained stable during the study (2 [1-3] days). The most common CMC subgroup across the study period were those with 1 non-NI CCC (13 303 [45.2%] of all CMC hospitalizations). Overall, 11 717 (39.8%) of CMC hospitalizations were among children assisted by a medical technology.

**Table 1.** Respiratory Admissions for Children With and Without Medical Complexity by Year

	Children with medical complexity					Children without medical complexity				
	2017	2018	2019	2020	2021	2017	2018	2019	2020	2021
Estimated population of children <18 y in Canada (excluding Quebec)	52 788	53 054	53 328	53 566	53 360	5 515 574	5 543 393	5 572 024	5 596 906	5 575 362
Respiratory hospitalization characteristics										
Hospitalization events, No.	7853	7353	6848	3275	4132	29 733	30 115	28 122	5422	16 225
Length of stay, median (IQR), d	5 (2-11)	5 (2-12)	5 (2-13)	6 (2-18)	5 (2-14)	2 (1-3)	2 (1-3)	2 (1-3)	2 (1-3)	2 (1-3)
Respiratory etiology, No. (%)										
Infectious	4365 (55.6)	4239 (57.6)	3858 (56.3)	1301 (39.7)	2026 (49.0)	20 078 (67.5)	20 606 (68.4)	19 187 (68.3)	2978 (54.9)	10 231 (63.1)
Noninfectious	3488 (44.4)	3114 (42.4)	2990 (43.7)	1974 (60.3)	2106 (51.0)	9655 (32.5)	9509 (31.6)	8935 (31.8)	2444 (45.1)	5994 (36.9)
ICU admission, No. (%)	2461 (31.3)	2335 (31.8)	2236 (32.7)	1333 (40.7)	1562 (37.8)	2001 (6.7)	2201 (7.3)	2046 (7.3)	465 (8.6)	1355 (8.4)
Hospitalization by province/territory, No. (%)										
British Columbia	796 (10.1)	794 (10.8)	678 (9.9)	380 (11.6)	511 (12.4)	4002 (13.5)	3889 (12.9)	3329 (11.8)	713 (13.2)	98 (0.6)
Alberta	1755 (22.3)	1415 (19.2)	1246 (18.2)	598 (18.3)	740 (17.9)	4903 (16.5)	4601 (15.3)	4553 (16.2)	860 (15.9)	2407 (14.8)
Saskatchewan	287 (3.7)	358 (4.9)	308 (4.5)	121 (3.7)	178 (4.3)	2240 (7.5)	2401 (8.0)	2141 (7.6)	419 (7.7)	2918 (18.0)
Manitoba	350 (4.5)	279 (3.8)	329 (4.8)	143 (4.4)	166 (4.0)	1021 (3.4)	1103 (3.7)	1213 (4.3)	253 (4.7)	1109 (6.8)
Ontario	4202 (53.5)	4041 (55.0)	3954 (57.7)	1891 (57.7)	2374 (57.5)	14 554 (48.9)	15 078 (50.1)	14 241 (50.7)	2650 (48.9)	8083 (49.8)
New Brunswick, Nova Scotia, Prince Edward Island, Newfoundland/Labrador	433 (5.5)	431 (5.8)	302 (4.4)	121 (3.7)	145 (3.5)	2701 (9.1)	2660 (8.8)	2390 (8.5)	427 (8.0)	1250 (8.1)
Northwest Territories, Nunavut, Yukon	30 (0.4)	35 (0.5)	31 (0.5)	21 (0.6)	18 (0.4)	312 (1.0)	383 (1.3)	255 (0.9)	100 (1.8)	98 (0.6)
Child characteristics										
Sex, No. (%)										
Male	4391 (55.9)	4018 (54.6)	3705 (54.1)	1885 (57.6)	2292 (55.5)	17 351 (58.4)	17 190 (57.1)	16 405 (58.4)	3093 (57.0)	9620 (59.3)
Female	3462 (44.1)	3335 (45.4)	3143 (45.9)	1390 (42.4)	1840 (44.5)	12 382 (41.6)	12 925 (42.9)	11 717 (41.6)	2329 (43.0)	6605 (40.7)
Age category										
<2 y^a^	2662 (33.9)	2602 (35.4)	2372 (34.6)	1211 (37.0)	1424 (34.5)	15 612 (52.5)	15 957 (53.0)	14 281 (50.8)	2478 (45.7)	8324 (51.3)
2-4 y	1878 (23.9)	1768 (24.0)	1667 (24.3)	574 (17.5)	1009 (24.4)	7760 (26.1)	7969 (26.5)	7588 (27.0)	1206 (22.2)	4981 (30.7)
5-9 y	1601 (20.4)	1440 (19.6)	1317 (19.2)	478 (14.6)	622 (15.1)	3, 905 (13.1)	3906 (13.0)	3928 (14.0)	807 (14.9)	1646 (10.1)
10-14 y	1010 (12.9)	885 (12.0)	867 (12.7)	520 (15.9)	557 (13.5)	1528 (5.1)	1445 (4.8)	1498 (5.3)	512 (9.4)	725 (4.5)
15-17 y	702 (8.9)	658 (8.9)	625 (9.1)	492 (15.0)	520 (12.6)	928 (3.1)	838 (2.8)	827 (2.9)	419 (7.7)	549 (3.4)
Mortality during respiratory hospitalization, No. (%)	188 (2.4)	163 (2.2)	187 (2.7)	114 (3.5)	129 (3.1)	11 (0.0)	5 (0.0)	5 (0.0)	<5	<5
Categorization of children with medical complexity, No. (%)										
NI and ≥1 CCC	1302 (16.5)	1191 (16.2)	1098 (16.0)	710 (21.7)	740 (17.9)	NA	NA	NA	NA	NA
Multiple CCC (excluding NI)	2159 (27.5)	1983 (27.0)	1884 (27.5)	1026 (31.3)	1117 (27.0)	NA	NA	NA	NA	NA
NI alone	794 (10.1)	799 (10.9)	676 (9.9)	276 (8.4)	403 (9.8)	NA	NA	NA	NA	NA
1 Non-NI CCC	3598 (45.8)	3380 (46.0)	3190 (46.6)	1263 (38.6)	1872 (45.3)	NA	NA	NA	NA	NA
Technology dependence, No. (%)	3038 (38.7)	2833 (38.5)	2633 (38.4)	1473 (45.0)	1740 (42.1)	NA	NA	NA	NA	NA

Abbreviations: CCC, complex chronic condition; ICU intensive care unit; NA, not applicable; NI neurologic impairment.

^a^ Excludes newborn admissions.

### Respiratory Hospitalizations

**Figure 1.**
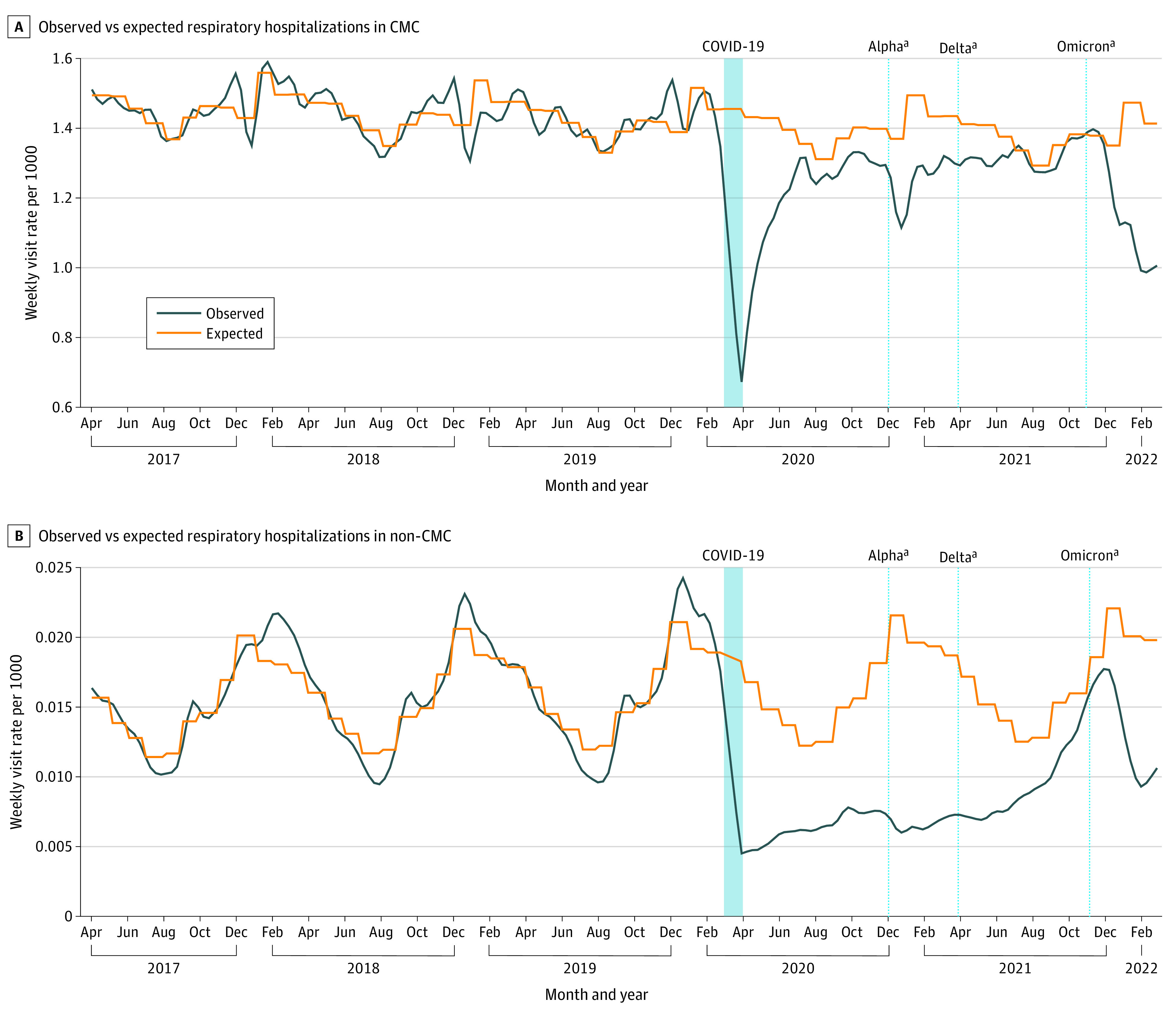
Observed vs Expected Respiratory Hospitalizations in Children With Medical Complexity (CMC) and Children Without Medical Complexity (Non-CMC) Weekly observed and expected counts based on children with and without medical complexity aged 0 to 17 years of age during prepandemic (April 1, 2017, to February 28, 2020) and pandemic (April 1, 2020 to February 28, 2022) periods. Shaded region indicates 1-month washout period (March 2020). ^a^Variant of concern emergence (Alpha, Delta, Omicron) based on first case in Canada.^32^

A comparison of observed and expected respiratory hospitalizations among CMC and non-CMC is summarized in Figure 1.^32^ Among CMC, compared with prepandemic annual respiratory hospitalization rates of 1385.6 per 10 000, hospitalizations in FY 2020 decreased to 611.4 per 10 000 CMC, corresponding to an annual rate difference of 774.2 per 10 000 CMC and a rate ratio (RR) of 0.44 (95% CI, 0.42-0.46) (Table 2). In FY 2021, hospitalizations decreased to 774 per 10 000 CMC, which corresponded to an annual rate difference of 611 per 10 000 CMC and an RR of 0.56 (95% CI, 0.51-0.62). Among non-CMC, there was an even larger relative reduction in respiratory hospitalizations in FY 2020 compared with prepandemic, decreasing from 52.9 per 10 000 in the prepandemic period to 9.7 per 10 000 in FY 2020, corresponding to an annual rate difference of 43.2 per 10 000 non-CMC, and a RR of 0.18 (95% CI, 0.17-0.19). Respiratory hospitalizations also decreased for non-CMC in FY 2021, with a comparable relative reduction to that observed in CMC (RR, 0.55 [95% CI, 0.54-0.56]). The absolute reduction during the pandemic was 7409 respiratory admissions for CMC and 37 448 for non-CMC.

**Table 2.** Serious Respiratory Illnesses in Children With and Without Medical Complexity Comparing Pandemic (2020-2021) to Prepandemic (2017-2019) Periods in Canadian Hospitals (Excluding Quebec)

Outcome	Children with medical complexity			Children without medical complexity		
	2017-2019	2020	2021	2017-2019	2020	2021
Respiratory hospitalization rate per 10 000 (95% CI)	1385.6 (1348.4-1422.8)	611.4 (586.7-636.1)	774.4 (746.6-802.2)	52.9 (52.6-53.2)	9.7 (9.4-9.9)	29.1 (28.7-29.6)
Rate ratio (95% CI)	1 [Reference]	0.44 (0.42-0.46)	0.56 (0.51-0.62)	1 [Reference]	0.18 (0.17-0.19)	0.55 (0.54-0.56)
Rate difference per 10 000 (95% CI)	[Reference]	774.2 (719.7-828.7)	611.2 (532.7-659.7)	[Reference]	43.2 (30.3-56.1)	23.8 (14.2-33.4)
Respiratory ICU admission rate per 10 000 (95% CI)	441.8 (431.5-452.2)	248.9 (235.7-262.6)	292.7 (278.4-307.6)	3.8 (3.7-3.9)	0.8 (0.7-0.9)	2.4 (2.3-2.6)
Rate ratio (95% CI)	1 [Reference]	0.56 (0.53-0.59)	0.66 (0.63-0.70)	1 [Reference]	0.22 (0.20-0.24)	0.65 (0.61-0.69)
Rate difference, per 10 000 (95% CI)	[Reference]	192.9 (165.7-220.1)	149.1 (125.2-173.0)	[Reference]	3.0 (0-6.4)	1.4 (0-3.7)
Respiratory mortality rate, per 10 000 (95% CI)	33.8 (31.0-36.8)	21.2 (17.6-25.6)	24.2 (20.2-28.7)	NA^a^	NA^a^	NA^a^
Rate ratio (95% CI)	1 [Reference]	0.63 (0.51-0.77)	0.72 (0.59-0.87)	NA^a^	NA^a^	NA^a^
Rate difference, per 10 000 (95% CI)	[Reference]	12.6 (5.6-19.6)	9.6 (3.5-15.7)	NA^a^	NA^a^	NA^a^

Abbreviations: ICU intensive care unit; NA, not available.

^a^ Cell sizes too small to provide stable estimates.

### Respiratory ICU Admissions

There was a similar pattern of reduced respiratory ICU admissions in both FY 2020 and FY 2021 for CMC compared with prepandemic. Respiratory ICU admissions for CMC decreased from 441.8 per 10 000 prepandemic to 248.9 per 10 000 (RR, 0.56 [95% CI, 0.53-0.59]) in FY 2020 and 292.7 per 10 000 (RR, 0.66 [95% CI, 0.63-0.70]) in FY 2021. For non-CMC, respiratory ICU admissions prepandemic were 3.8 per 10 000 with a reduction to 0.8 per 10 000 (RR, 0.22 [95% CI, 0.20-0.24]) in FY 2020 and 2.4 per 10 000 (RR, 0.65 [95% CI, 0.61-0.69]) in FY 2021. The absolute reduction of respiratory ICU admissions was 1829 for CMC and 2460 for non-CMC during the pandemic period.

### Mortality During Respiratory Hospitalization

Among CMC, compared with prepandemic (33.8 per 10 000), mortality during respiratory hospitalizations decreased in both FY 2020 (21.2 per 10 000; RR, 0.63 [95% CI, 0.51-0.77]) and FY 2021 (24.2 per 10 000; RR, 0.72 [95% CI, 0.59-0.87]). Mortality was not assessed in the non-CMC population as the incidence was too low to provide stable estimates. The absolute reduction of in-hospital deaths from respiratory illness was 119 among CMC during the pandemic period.

### Additional Analyses

**Figure 2.**
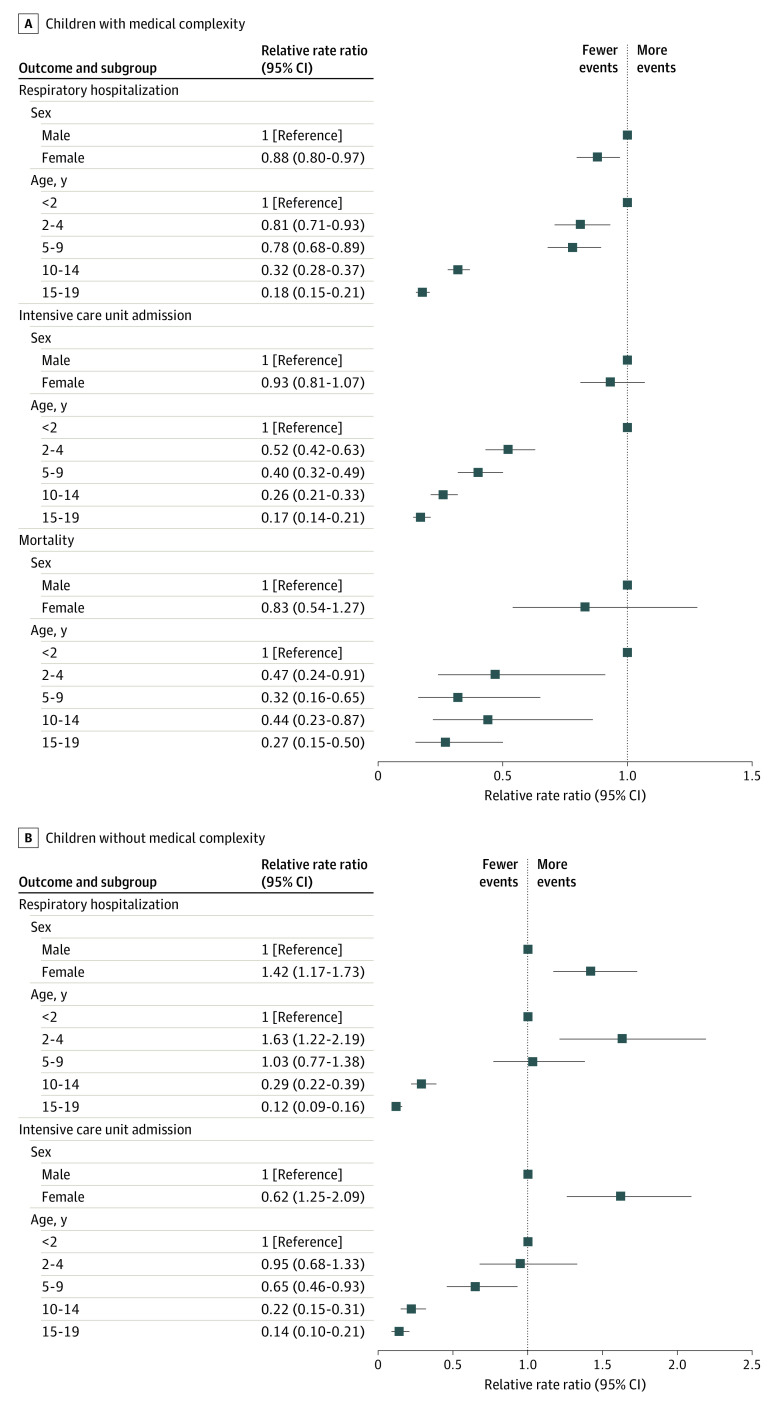
Relative Rate Ratios of Respiratory Hospitalization, Intensive Care Unit Admission, and Mortality Comparing Pandemic and Prepandemic Periods by Sex and Age Relative rate ratios (95% CI) of respiratory hospitalization, intensive care unit admission, and mortality comparing pandemic and prepandemic periods by sex and age categories for (A) children with medical complexity (CMC) and (B) children without medical complexity (non-CMC).

In stratified analyses, among CMC, female children had a larger reduction of respiratory hospitalizations compared with male children (relative rate ratio [RRR], 0.88 [95% CI, 0.80-0.97]), and the same pattern was observed among all groups of children greater than 2 years of age compared with those aged less than 2 years (Figure 2). Among non-CMC, there was a smaller reduction of respiratory hospitalizations among female children vs male children (RRR, 1.42 [95% CI, 1.17-1.73]) and among children aged 2 to 4 years vs those aged younger than 2 years (RRR, 1.63 [95% CI, 1.22-2.19]). A larger relative reduction in these events was observed in children at least 10 years of age.

### Sensitivity Analysis

When limiting respiratory hospitalizations to those from infectious causes, a similar pattern of decreased relative rates was observed (CMC in FY 2021: RR, 0.31 [95% CI, 0.29-0.33]; CMC in FY 2021: RR, 0.48 [95% CI, 0.46-0.50]; non-CMC in FY 2020: RR, 0.15 [95% CI, 0.14-0.16]; non-CMC in FY 2021: RR, 0.51 [95% CI, 0.48-0.54]) (eTable 4 in Supplement 1). Among CMC, mortality during respiratory hospitalizations from infectious causes decreased in FY 2020 (RR, 0.45 [95% CI, 0.26-0.77]), but not in FY 2021 (RR, 0.95 [95% CI, 0.64-1.41]).

## Discussion

In this cross-sectional study, we observed decreased respiratory-related hospitalizations, ICU admissions, and mortality during the first 2 years of the pandemic. The relative reduction in acute care use for respiratory illnesses was more substantial among non-CMC than CMC in the first year of the pandemic, but similar in both groups in the second pandemic year. Similar findings were observed when limiting analysis to respiratory hospitalizations with an infectious diagnosis, except for mortality for CMC in FY 2021. Taken together, this degree of serious respiratory illness reduction over the 2 pandemic years corresponds to a decrease in over 44 500 hospitalizations among Canadian children (7409 for CMC, 37 448 for non-CMC), over 4200 ICU admissions (1829 for CMC, 2460 for non-CMC) and a decrease of 119 CMC in-hospital deaths.

Our study expands on previous reports of decreased overall pediatric hospital use during the pandemic. A single center study from Israel reported a comparable 60% reduction in hospitalizations during lockdown, but did not detect differences among individuals with and without preexisting conditions, which may be because they focused solely on a short, intense lockdown period.^33^ A larger multicenter study of children’s hospitals in the United States evaluating the early period of the pandemic reported a 14.4% decrease in all-cause hospitalizations among children with NI.^11^ Another multicenter study of children’s hospitals in the United States evaluated the first year of the pandemic among CMC and reported a 20% decline in all-cause hospitalizations but did not observe a decline in ICU use.^10^ Our study focused specifically on respiratory hospitalizations which may have been associated with greater pandemic-era declines than hospitalizations overall, and extended evaluation to a longer pandemic period, focused on broader groups of CMC, and included all hospital admissions, not just those in children’s hospitals.

The findings of greater relative mitigation of respiratory hospitalizations in non-CMC compared with CMC in FY 2020 was surprising as we expected greater declines among CMC due to their elevated risk. Potential explanations for this observation include the ongoing circulation of other respiratory viruses during the pandemic for which CMC are at particular risk for hospitalization (eg, enterovirus),^34^ unavoidable respiratory admissions unrelated to an infection (eg, noninfectious triggers for asthma or aspiration), and the use of nonpharmacologic infection-prevention strategies to reduce infection risk prepandemic among families of CMC. These explanations may also be relevant in understanding why older children who have a baseline lower risk of respiratory hospitalizations^35^ were also observed to have greater relative decreases in respiratory admissions during the pandemic. It is important to emphasize that despite the larger attenuation of respiratory hospitalizations among non-CMC in the first pandemic year, given the much higher baseline prevalence of CMC respiratory hospitalizations, the decline observed among CMC is clinically important and was associated with decreased mortality.

### Strengths and Limitations

To our knowledge, this study is the longest evaluation (2-year pandemic period) comparing CMC with non-CMC respiratory hospitalizations using population-level data published to date. Nevertheless, the study has limitations. First, although we used an algorithm for ascertaining CMC that has been used extensively in Canadian health services research,^27^ administrative data are unable to capture important domains of complexity such as family and/or caregiver needs, psychosocial complexity, and functional status; and administrative data were limited to those with previous hospitalization data. Second, we used PECCS respiratory codes that excluded admissions for underlying respiratory conditions that are likely unrelated to viral infections (eg, bronchopulmonary dysplasia). Among the included codes were diagnoses for which hospitalization may or may not be attributed to a viral respiratory infection (eg, asthma exacerbations by infectious or noninfectious triggers),^19^ although hospitalization and ICU admission rates did not change when these codes were excluded. Third, we limited capture of COVID-19 diagnoses to those with an additional PECCS respiratory code (eg, pneumonia). Although we may have missed some cases that were misclassified, at the time, 43.2% of Canadian children admitted to hospital with SARS-CoV-2 infections were not admitted because of COVID-19 (they typically had incidental SARS-CoV-2 infection detected during universal screening at hospital admission).^15^ Fourth, the data sets used did not include out-of-hospital mortality from respiratory illnesses; however, more than 80% of CMC deaths occur in hospital.^16^ Fifth, this study was conducted in Canada, which had less severe outcomes related to COVID-19 than the United States^36^; this may be due in part to more widespread adoption of public health measures or other factors. For instance, in Ontario, Canada’s most populous province, mandatory masking, daily symptom checks, social distancing, and cohorting were instituted in schools at the start of the 2020 to 2021 school year.^37^ Findings may differ in other jurisdictions. Additionally, this study cannot identify causative factors related to the reduction of hospitalization, ICU admissions, and mortality between CMC and non-CMC.

## Conclusions

In this cross-sectional study, we observed decreased hospitalizations and ICU admissions related to respiratory illnesses for both CMC and non-CMC during the COVID-19 pandemic and decreased in-hospital mortality among CMC. This study’s results suggest that the outcomes of public health interventions are not always equal across population groups. Groups of people with greater risk require special attention and monitoring when crafting population-level recommendations. Future evaluations of the effect of nonpharmaceutical interventions during subsequent periods in the pandemic when the infection rate in children was higher (eg, Omicron) and during nonpandemic periods of increased respiratory illness may be warranted.

## Notes

Supplement 1. **eTable 1.** Public Policy Interventions Instituted in Canada During the COVID-19 Pandemic **eTable 2.** Description of the Canadian Institute of Health Information Discharge Abstract Database Used in the Study **eTable 3.** Pediatric Clinical Classification System (PECCS) **eTable 4.** Serious Respiratory Illnesses in Children With and Without Medical Complexity Comparing Pandemic (2020, 2021) to Pre-Pandemic (2017-2019) Periods in Canadian Hospitals (Excluding Quebec) Limiting to Those Illnesses With an Infectious Diagnosis

Supplement 2.
Data Sharing Statement
