# Supplementary material for: Severe Respiratory Disease Among Children With and Without Medical Complexity During the COVID-19 Pandemic
Source: JAMA Netw Open. 2023 Nov 14;6(11):e2343318. doi: 10.1001/jamanetworkopen.2023.43318 (PMC10646732; doi:10.1001/jamanetworkopen.2023.43318)
Supplement: Supplement 1. — eTable 1. Public Policy Interventions Instituted in Canada During the COVID-19 Pandemic eTable 2. Description of the Canadian Institute of Health Information Discharge Abstract Database Used in the Study eTable 3. Pediatric Clinical Classification System (PECCS) eTable 4. Serious Respiratory Illnesses in Children With and Without Medical Complexity Comparing Pandemic (2020, 2021) to Pre-Pandemic (2017-2019) Periods in Canadian Hospitals (Excluding Quebec) Limiting to Those Illnesses With an Infectious Diagnosis [file jamanetwopen-e2343318-s001.pdf]

Supplemental Online Content

Belza C, Pullenayegum E, Nelson KE, et al. Severe respiratory disease among children with and without medical complexity during the COVID-19 pandemic. *JAMA Netw Open.* 2023;6(11):e2343318. doi:10.1001/jamanetworkopen.2023.43318

eTable 1. Public Policy Interventions Instituted in Canada During the COVID-19 Pandemic

eTable 2. Description of the Canadian Institute of Health Information Discharge Abstract Database Used in the Study

eTable 3. Pediatric Clinical Classification System (PECCS)

eTable 4. Serious Respiratory Illnesses in Children With and Without Medical Complexity Comparing Pandemic (2020, 2021) to Pre-Pandemic (2017-2019) Periods in Canadian Hospitals (Excluding Quebec) Limiting to Those Illnesses With an Infectious Diagnosis

This supplemental material has been provided by the authors to give readers additional information about their work.

© 2023 Belza C et al. *JAMA Network Open.*

eTable 1. Public Policy Interventions instituted in Canada during the COVID-19 Pandemic

Based on the Oxford Covid-19 government response tracker (OxCGRT) indicators. There was substantial variation across provinces and territories, as well as over time, in the implementation of non-pharmaceutical interventions in Canada.

OxCGRT Indicators	
Containment and Closure	• School closure<br>• Workplace closure<br>• Cancelation of public events<br>• Restrictions on gathering size<br>• Close public transit<br>• Stay at home requirements<br>• Restrictions on internal movement<br>• Restrictions on international travel
Economic Response	• Income support<br>• Debt/contract relief for households<br>• Fiscal measures<br>• Giving international support
Health Systems	• Public health campaigns<br>• Testing policy<br>• Contact tracing<br>• Emergency investment in healthcare<br>• Facial coverings<br>• Vaccination policy

Hale T, Angrist N, Goldszmidt R, et al. Variation in the Canadian Provincial and Territorial responses to COVID-19. Blavatnik School of Government Working Paper, 2021. Available: www.bsg.ox.ac.uk/covidtracker. Accessed September 28, 2023.

© 2023 Belza C et al. *JAMA Network Open.*

eTable 2. Description of the Canadian Institute of Health Information Discharge Abstract Database Used in the Study

Data Source	Description
**Canadian Institutes of Health Information Discharge Abstract Database (CIHI-DAD)**	The Canadian Institutes of Health Information Discharge Abstract Database (CIHI-DAD) contains administrative, clinical (diagnoses and procedures/interventions), demographic, and administrative information from all admissions to acute care hospitals in Canada. Provinces/territories (excluding Quebec) require reporting from all facilities. Data related to diagnoses and interventions are coded using ICD-10-CA. The DAD includes data reported from 591 acute care institutions. For the current study, data were available for all respiratory hospitalizations outside the province of Quebec, comprising approximately 77% of Canada's population. CIHI receives records from hospitals, validates and cleans them.

© 2023 Belza C et al. *JAMA Network Open.*

eTable 3. Pediatric Clinical Classification System (PECCS)
PECCS codes were developed using US administrative pediatric hospitalization data algorithms.

Respiratory Illness Diagnoses	PECCS Grouper Code	ICD-10
Infectious		
Pneumonia	90010	A202, A212, A221, A310, A370, A371, A430, A481, B052, B250, B371, B380, B381, B382, B390, B391, B392, B485, B583, B59, J100, J110, J120, J121, J122, J123, J128, J129, J13, J14, J150, J151, J152, J153, J154, J155, J156, J157, J158, J159, J160, J168, J170, J171, J172, J173, J178, J180, J181, J188, J189, J851
Acute Bronchiolitis	125001	J210, J211, J218, J219
Influenza	123000	J09, J101, J108, J111, J118
Common Cold	126004	J00
Croup	126007	J042, J050
Lung Abscess	133002	J852
Empyema/ Pyothorax	130001	J860, J869
Acute upper respiratory infection	126010	J040, J060, J068, J069
Acute bronchitis	125000	J200, J201, J202, J203, J204, J205, J206, J207, J208, J2080, J2088, J209
Pertussis	003001	A378, A379
Viral infection	007000	B970, B971, B972, B973, B974, B975, B976, B977, B978, B9780, B9781, B9788
Non-Infectious/ Generic		
Asthma	128000	J4500, J4501, J4510, J4511, J4580, J4581, J4590, J4591
Aspiration pneumonitis; food/ vomitus	129000	J690
Other lower respiratory disease	133000	J182, J22, J81, J82, J840, J841, J849, J853, J984, J986, J988, J989, J990, J991, J998, R042, R048, R049, R063, R064, R066, R068, R071, R093, R91, Z870, Z876, Z942
Respiratory failure; insufficiency; arrest	131000	J80, J960, J9600, J9601, J9609, J961, J9610, J9611, J9619, J969, J9690, J9691, J9699, R090, R092, Z991

© 2023 Belza C et al. *JAMA Network Open.*

eTable 4. Serious Respiratory Illnesses in Children with and without Medical Complexity Comparing Pandemic (2020, 2021) to Pre-Pandemic (2017-2019) Periods in Canadian Hospitals (excluding Quebec) Limiting to those Illnesses with an Infectious Diagnosis

	**Children with Medical Complexity**			**Children without Medical Complexity**		
	**2017-2019**	**2020**	**2021**	**2017-2019**	**2020**	**2021**
Respiratory Infection Admission, rate per 10,000 (95% CI)	782.9 (769.3-796.8)	242.9 (229.9-256.4)	379.7 (363.3-396.6)	36.0 (35.7-36.3)	5.3 (5.1-5.5)	18.4 (18.0-18.7)
Rate Ratio (95% CI)	Ref	0.31 (0.29-0.33)	0.48 (0.46-0.50)	Ref	0.15 (0.14-0.16)	0.51 (0.48-0.54)
Absolute Rate Reduction, per 10,000 (95% CI)	Ref	540.0 (494.5-585.5)	403.2 (363.8-442.6)	Ref	30.7 (19.8-41.6)	17.6 (9.4-25.8)
Respiratory Infection ICU admission, rate per 10,000 (95% CI)	154.4 (148.4-160.6)	59.7 (53.2-66.3)	87.7 (79.8-95.7)	2.1 (2.0-2.2)	0.2 (0.2-0.3)	1.2 (1.1-1.3)
Rate Ratio (95% CI)	Ref	0.39 (0.27-0.51)	0.57 (0.47-0.69)	Ref	0.11 (0-0.28)	0.58 (0.50-0.66)
Rate Difference, per 10,000 (95% CI)	Ref			Ref		
Respiratory Infection Mortality, rate per 10,000 (95% CI)	6.3 (5.1-7.5)	2.8 (1.4-4.2)	6.0 (3.9-8.1)	*	*	*
Rate Ratio (95% CI)	Ref	0.45 (0.26-0.77)	0.95 (0.64-1.41)	*	*	*
Absolute Rate Reduction, per 10,000 (95% CI)	Ref	3.5 (0-7.2)	0.3 (0-1.4)	*	*	*

Abbreviations: ICU intensive care unit
* Cell sizes too small to provide stable estimates

© 2023 Belza C et al. *JAMA Network Open.*
