# Supplementary material for: Severe Respiratory Disease Among Children With and Without Medical Complexity During the COVID-19 Pandemic
Source: JAMA Netw Open. 2023 Nov 14;6(11):e2343318. doi: 10.1001/jamanetworkopen.2023.43318 (PMC10646732; doi:10.1001/jamanetworkopen.2023.43318)
Supplement: Supplement 2. — Data Sharing Statement [file jamanetwopen-e2343318-s002.pdf]

Data Sharing Statement

Belza. Severe Respiratory Disease Among Children With and Without Medical Complexity During the COVID-19 Pandemic. *JAMA Netw Open*. Published November 14, 2023.
doi:10.1001/jamanetworkopen.2023.43318

Data
Data available: No

Additional Information
Explanation for why data not available: The data are owned by the Canadian Institute of Health Information.
